# Protein hydrolysates from fish wastes: nutritional characteristics and its inclusion in diets for *Octopus maya*

**DOI:** 10.1371/journal.pone.0321572

**Published:** 2025-04-18

**Authors:** Honorio Cruz-López, Cristina Pascual, Magalli Sanchez, Pedro Domingues, Carlos Rosas, Pedro Gallardo

**Affiliations:** 1 Unidad Multidisciplinaria de Docencia e Investigación, Facultad de Ciencias, Universidad Nacional Autónoma de México, Sisal, Yucatán, México; 2 Instituto Español de Oceanografía, Centro Oceanográfico de Vigo, Vigo, Spain; Universiti Malaysia Kelantan, MALAYSIA

## Abstract

The utilization of fish waste protein as an alternative to crab and squid protein presents an important alternative for octopus fattening. During this study, nutritional characteristics of fish protein hydrolysate (FPH) and its inclusion in prepared diets were evaluated on growth performance and enzyme activity of digestive gland of *O. maya* juveniles. FPH were prepared using fish waste and their nutritional properties were evaluated. Four diets with different levels of FPH (0%, 10%, 15%, and 20%) in substitution for crab meals were fed to octopuses (mean body weight 100 mg) individually distributed for 70 days. Regarding yield, at the end of the hydrolysis period (day 15) the FPH fraction constitutes 67% of the total silage (dried powder). Small peptides were recorded in FPH (< 2.12 DA). Altogether, 17 amino acids were identified on FPH, encompassing nine essential amino acids (EAAs; 182 mg g^-1^) and eight non-essential amino acids (NEAAs; 427 mg g^-1^). Also, the free amino acids (FAAs) content was 8.3% of the total amino acids content with the predominance of taurine. Octopuses fed with FPH15 had the highest weight gain (3.06 g), SGR (4.76% day^-1^), and survival (90%) compared to FPH0. Total alkaline protease activity of octopuses digestive gland was lower in FPH20 (3550 U mg of protein^−1^) than in the control (5277 U mg of protein^−1^). Incorporating protein hydrolysate derived from fish waste into prepared diet may offer unique advantages in promoting optimal growth and general physiological well-being for *O. maya*.

## Introduction

Growing interest in fish consumption has produced an increase in fish production. Therefore, valorization of fish by-products is becoming increasingly relevant, as marketing moves from selling whole fish to filleting [1]. By-products account for around 60–70% of the live fish weight of most commercially processed fish; without the right management, environmental problems may occur [2]. Furthermore, these wastes provide a useful source of bio-functional and nutritional compounds, such as protein, lipids, minerals, vitamins, and enzymes [2]. They contain high-quality protein (15–30% wet base) with all essential amino acids and can be used as feed ingredients, and especially as a substrate for hydrolysates production [3,4]. Due to its low cost and environmental friendliness, ensiling is one of the best methods to produce FPH. Silage is produced by endogenous proteases existing in fish waste which allows separation into phases; solid, fat and an aqueous phase with a high content of soluble protein, peptides, and free amino acids [5,6]. FPH is a mix of peptides, making them more digestible and attractive to animal feeds. They also play a significant role in metabolic regulation, modulation, and facilitate the molecules absorption by intestinal epithelial cells [4,7,8]. These hydrolysates are used as a protein supplement for *Channa striata* (45%), *Lates calcarifer* (5–10%), *Pseudosciaena crocea* (10%), *Paralichthys olivaceus* (2.3–11%), and *Penaeus vannamei* (6%), and boosted growth, feed efficiency, immune parameters, intestinal health, and disease resistance [9–12]. In cephalopods, Le Bihan et al. [13] used protein hydrolysates (< 6.5 DA) in diets for juvenile cuttlefish (*Sepia officinalis*), with supplementation contributing to healthy, fast growth and higher survival.

The four-eyed octopus (*Octopus maya*) is an integral part of food security, supporting human health and socio-economic development in the Yucatan Peninsula, Mexico. Owing to its direct development, fast growth, short life cycle, easy adaptation to captivity and high international market value, *O. maya* is recognized as an innovative species for aquaculture [14,15]. *O. maya* is a carnivorous species and large protein and amino acid contents in the diet are needed to fulfill energy demands [16]. Therefore, protein is the most important and costly component in octopus feeds. Previous studies have focused on the nutritional effect of several protein source in diets for *O. maya*; concerning growth and feed conversion, the best performances were obtained with diets composed of crab (*Callinectes sapidus*) or mixed (squid/crab) [17–23]. Thus, diets with 70% squid meal (*Dosidicus gigas*) and 30% blue crab meal are currently the best for *O. maya*, regarding costs [19]. However, there are some concerns about using squid/crab protein in octopus diets, mainly related to costs and availability. Therefore, it is necessary to find ingredients or food additives that improve the performance and functionality of diets to obtain higher production of octopus at a commercial level.

Currently, the “Moluscos del Mayab” cooperative farmers octopuses on a pilot scale in the port of Sisal, Yucatán. Consequently, this farmer currently demands sustainable protein ingredients that can compete with crab and squid meals regarding nutritional quality and economic feasibility; therefore, the exploration of feed ingredients from marine biowastes plays a pivotal role for octopus fattening. In our previous study, silage from fish by-products showed a high protein content with an adequate balance in amino acids, which makes it an attractive source of not only protein, but also functional peptides (unpublished data). Therefore, fish hydrolysate derived aqueous phase could have peptides with different molecular weight that promote *O. maya* growth and health. To our knowledge, no information on the effects of FPH supplementation on *O. maya* juveniles, whose digestive features suggest the need for easily digestible food, has been reported. Thus, the present research could provide information for a potential solution to problems of the ingredients supply for octopus. Therefore, the main goals of this study were to determine the nutritional composition of fish protein hydrolysate and subsequently, the effects of the inclusion of protein hydrolysates in diets for *O. maya* juveniles on growth and physiological condition.

## Materials and methods

### Ethical statement

This research was conducted under the Ethics and Scientific Responsibility Commission (CEARC) of the Facultad de Ciencias at Universidad Nacional Autonoma of Mexico, permit approval no. 25102021. No specific collection permits were required given that octopus juveniles were provided by Laboratorio de Ecofisiología Aplicada of the Unidad Multidisciplinaria de Docencia e Investigación (UMDI-UNAM). The octopuses were sedated with cold seawater (15 °C) and euthanized by brain puncture [24,25]. Moreover, a strong effort was directed to minimize animals killing, and the minimum necessary number of animals involved in the experiments.

### Fish hydrolysate preparation and characterization

**Fig 1:**
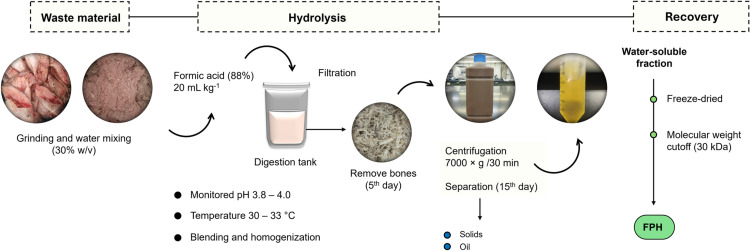
Flow chart of fish protein hydrolysate production.

Fish wastes were obtained from the Tigres del Mar Fishermen Cooperative, (Sisal, Yucatan, Mexico) from September-October 2023. They consisted of white grunt (*Haemulon plumierii*) heads, frames, trimmings, and guts. Fish protein hydrolysate was produced by autolytic hydrolysis process facilitated by the action of the endogenous enzymes present in the fish waste. The wastes were mixed with water at the rate of 30% (w/v) and homogenized using an industrial blender (LM-12, Torrey, Mexico) [26]. The minced samples were then divided into three portions (5 kg each part) and placed in plastic containers (20 L) followed by the addition of formic acid (88%) at the rate of 20 mL kg^-1^ to reduce the pH to 3.8–4.0 according to Gallardo et al. [26]. Butylated hydroxytoluene (BHT) was added at a rate of 0.02 mg 100 g of fat as an antioxidant and kept at room temperature (30–33 °C). The samples were homogenized daily, and the pH was measured with a digital pH meter and adjusted to pH 3.8–4.0 Fig 1. After 5 days of ensiling the samples were filtered through two layers of cheesecloth to remove undigested material (bones). The hydrolysis process was monitored, and samples were collected for protein and SDS-PAGE analysis. After 15 days, the hydrolysate obtained was used for the separation of fractions by centrifugation at 7000 × g at 20 °C for 30 min. The water-soluble fraction collected after centrifugation was categorized as FPH. Finally, the FPH was freeze-dried (Labconco FreeZone 12, Labcono Corporation) and stored at −20 °C. The degree of hydrolysis estimation was determined by formal titration according to Noman et al. [27]. The total nitrogen was determined following the Kjeldahl method.

### Soluble protein and SDS-PAGE

Gel electrophoresis analysis was done according to the method of Haider et al. [28]. The samples was mixed at a 1:2 (v/v) ratio with sample buffer (0.1 M Tris-HCl, pH 6.8, containing 1% SDS, 20% glycerol, 4% β-ME and 0.02% Coomassie blue) and heated to 100 °C for 5 min and samples were run on a acrylamide gel (4% stacking and 10% separating) using Tricine-SDS-PAGE until the dye front reached the end of the gel. The gel was stained with Coomassie solution for one hour and then destained with water until clear bands were visible.

### Molecular weight distribution

The dried sample was dissolved in ultrapure water at 30 mg mL^-1^ and fractionated through membrane of molecular weight cutoff (MWCO 30 DA) to separate small peptides. The filtrate was recovered, and the molecular weight distribution was analyzed by gel permeation chromatography (GPC) using the column (Phenomenex-Yarra 3 μm SEC-2000 145 Å SEC 300 × 7.8 mm). The acetonitrile/water/trifluoroacetic acid 10/89.9/0.1(v/v) was used as the mobile phase at a flow rate of 1.0 mL min for 20 min, and the column temperature was 25 °C. The 20 μL of sample was injected into the liquid chromatography system and the absorbance was monitored at 215 nm. The average molecular weight of protein hydrolysate was calculated from the standard curve drawn of the standards, ovalbumin (45000 Da), ribonuclease (13700 Da), bacitracin (1420 Da), and dipeptide GG (132 Da), and the data were analyzed using the Agilent galaxies software.

### Proximate analysis

Chemical composition was determined by the standard official methods of the A.O.A.C. (Association of Official Analytical Chemists) [29]. The total nitrogen content was determined by the Kjeldahl method (method 928.08). The protein content of silage was obtained using the standard nitrogen to protein conversion factor of N × 6.25. Determination of moisture in silage was quantified using the gravimetric method (105 °C for 12 h) (method 950.46), and ash content was measured by combustion in a furnace at 550 °C (method 920.153). Total lipids were measured by modifying the Folch extraction method [30].

### Amino acid analysis

Samples were hydrolyzed with 6 N hydrochloric acid and 0.06% phenol and incubated in a nitrogen atmosphere at 113 °C for 18 h [31]. After hydrolysis, samples and standards were derivatized with OPA and FMOC (O-phthaldehyde: Fluorenylmethyloxycarbonyl) reagent and reconstituted in a sodium phosphate buffer. Finally, amino acids content was analyzed by HPLC system (Agilent Technologies, Santa Clara, CA, USA). Amino acid quantification was done using a standard amino acid mixture as reference and expressed as g 100g^-1^ dry sample.

### Experimental design

#### Diets.

The diet formulation was done following Gallardo et al. [19] Table 1. FPH0 (basic diet, 0% of FPH), FPH10 (10% of FPH), FPH15 (15% of FPH), and FPH20 (20% of FPH) were done to replace an equal percentage of crab meals. All ingredients were grinded into a fine powder, passed through a 250 µm mesh, and mixed in a kitchen Aid K45ss blender. The mixture was then pelletized with a meat grinder (Torrey M-12-FS, Mexico), dried at 55 °C for 1 h, packed in sealed bags, and kept at 4 ºC until use. Proximate analysis was performed as described previously.

**Table 1:** Ingredients (%) and analyzed composition of the diets.

Ingredients (g kg^-1^ of dry basis)	FPH0	FPH10	FPH15	FPH20
**Squid mantle meal**	43.5	43.5	43.5	43.5
**Crab meal**	43.5	33.5	28.5	23.5
**Fish Protein hydrolysate**	–	10	15	20
**Grenetin**	10	10	10	10
^**1**^ **Vitamin and mineral mix**	1	1	1	1
^**2**^ **Vitamin C (Stay C)**	2	2	2	2
**Chemical analysis (%)**				
**Moisture**	4.2	4	4.6	4.7
**Crude protein**	72	66	64	61
**Crude fat**	2.8	2.9	2.9	3.0
**Ash**	7.6	6.9	6.6	6.3
**NFE** ^*^	13.4	20.2	21.9	25

Squid mantle commercial (*D. gigas*).

Crab meal (*C. sapidus*).

^1^Vitamins and Minerals without vit C done by DSM of México. Mineral mix: Co, 2 g kg^−1^; Mn, 16 g kg^−1^; Zn, 40 g kg^−1^; Cu, 20 g kg^−1^; Fe, 0.001 g kg^−1^; Se, 0.1 g kg^−1^.

^2^Vitamin C: Stay-C® by DSM nutritional products L-ascorbyl-2-polyphospate, 35% active.

* Nitrogen-Free Extract (calculated by difference) = 100 – (protein + lipid + ash).

#### Feeding experiment.

A total of 80 octopuses (0.103 ± 0.004 g) were used and placed individually in plastic containers (1 L capacity) with mesh walls to allow adequate seawater circulation. A 1/2“ PVC elbow was placed in each plastic container as a shelter. Individualized octopuses were then randomly distributed in 4 rectangular tanks (1.5 x 1.2 x 0.4 m) (20 octopuses experimental diet). Octopus were fed with experimental diets for 70 days. Ammonia, nitrite, and nitrate concentrations were determined once a week in each tank using a kit AQ-4 (LaMotte, 363504). Temperature, salinity, pH, and dissolved oxygen were also recorded daily using a YSI Pro20 (Yellow Spring Instruments, Yellow Spring, OH, USA). Sea water quality parameters were recorded daily and maintained within the adequate ranges for this species [14]. In these conditions, dissolved oxygen was maintained between 5 and 6 mgL^‐1^, salinity at 36 ups, and temperature between 25 and 27 °C. Nitrate, nitrite, and ammonia were always lower than 10, 0.15 and 0.6 mg L^‐1^ respectively. Light was maintained at 75 lux cm^2^.

#### Growth and survival.

At the end of the feeding trial, octopuses were weighed in a glass container with seawater 5 seconds after being removed from the water, to allow more accurate measurements. The weight gain (WG), specific growth rate (SGR), average daily gain (ADG), survival rate, hepatosomatic index (HSI) were calculated using the following formulas:


WG= Final body weight, g − Initial body weight, g (1)

$$SGR\;({\%\;day}^{-1})\;=\;100\;x\;\left(\frac{In\;final\;body\;weight\;-\;In\;initial\;body\;weight}{number\;of\;days}\right)$$ (2)

$$Survival\;rate\;(\%)\;=\;100\;x\;\left(\frac{\;Final\;number\;of\;octopus}{Initial\;number\;of\;octopus}\right)$$ (3)

$$HSI\;(\%)\;=\;100\;x\;\left(\frac{Digestive\;gland\;weight,\;g}{Body\;weight,\;g}\right)$$ (4)

### Biochemical analyses of tissues

To obtain the different tissues, octopuses were sedated with the cold seawater bath (15 °C) for 1–2 min and euthanized by brain puncture [24,25]. The digestive gland (DG) and arms were removed, frozen in liquid nitrogen and stored at –80 °C until analysis. Glycogen in tissues was determined using the method described by Carroll et al. [32]. DG and arms (20–30 mg) were homogenized with trichloroacetic acid (TCA 5%) using Minilys-Personal Homogenizer (Bertin Technologies, Paris, France) and centrifuged at 4500 × g for 5 min. Subsequently 100 μL of supernatant was piped into a tube and mixed with 500 μL of ethanol 95%. Tubes were mixed and placed in an oven at 37 °C for 3 h. After, the tubes were centrifuged at 4550 × g for 15 min at 25 °C. The supernatant was discarded, leaving the glycogen as a pellet. By adding 1 mL concentrated sulfuric acid and 200 μL phenol 5%, glycogen was dissolved. From the mix, 200 μL was transferred to a microplate and read at 490 nm in microplate reader.

### Enzymatic activity in the digestive gland

DG sample was homogenized with cold pyrogen-free water (1:20, w/v) using a Minilys-Personal Homogenizer for extraction of enzymes. The samples were centrifuged at 10000 × g for 20 min at 4 °C. The supernatants (DG extracts) were stored at −80 °C and used for all enzyme analysis. Total soluble protein was measured by the method Bradford [33] using a commercial chromogen reagent (Bio-Rad, #500–0006). The activity of total proteolytic enzymes was determined as described by Anson [34]. Acid protease activity was assayed using hemoglobin (1% dissolved using Stauffer solution pH 3) as substrate. The enzyme extract and substrate were mixed (1:10), and then the tubes were incubated for 10 min at 37 °C. The reaction stopped with trichloroacetic acid at 20% and cooled for 15 min at 4 °C to allow protein precipitation. The extracts were centrifuged at 10000 × g for 15 min. The absorbance of the supernatants was measured by a spectrophotometer at 280 nm. The same procedure was used to determine alkaline peptidases activity, with casein at 1% as substrate at pH 8. All enzyme activities were calculated in the same way to unify units and thus expressed as U mg^-1^ protein, where one unit of enzyme activity was defined as the change of absorbance per min.

### Statistical analysis

Data were tested for normality (Kolmogorov–Smirnoff test) and homogeneity of variance (Levene’s test) at a significance level of 5% prior to further analysis. One-way analysis of variance (ANOVA) was performed, followed by Duncan’s test to compare mean values between treatments at (p < 0.05) using Statistica software® (version 10.0). All results are presented as mean ± standard deviation (SD).

## Results and discussion

### Characteristics of protein hydrolysate

#### Proteolysis and protein pattern.

**Fig 2:**
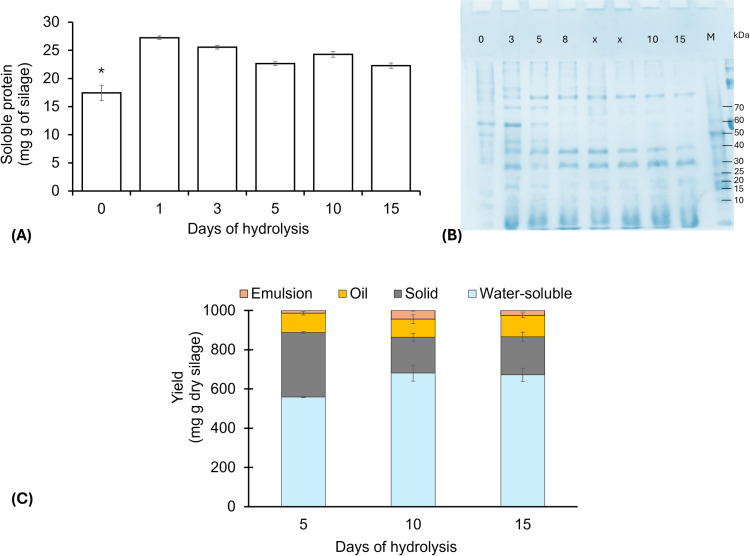
Soluble protein (A), protein pattern (B), and yield of protein hydrolysate (C) from fish waste. Lane M: protein marker; lane 0, 3, 5, 8, 10, and 15 days of hydrolysis; unused lanes are marked with an X.

The soluble protein was measured to monitor the progress of hydrolysis during ensiling over time. As seen in Fig 2A, after 24 h of hydrolysis (D1), the soluble protein content showed a sharp increase. This could be explained by sarcoplasmic proteins released during the proteolytic breakdown of tissue cells, which increases the concentration of proteins in the water-soluble fraction [6]. Subsequently on D3, a slight decrease was observed, indicating breakdown of the peptide bonds of polypeptides to yield smaller peptides and/or free amino acids. The degree of hydrolysis (DH) observed for the fish hydrolysate was 49.6 ± 2.0%, indicating the presence of enzymes naturally found in fish waste with high activity at acidic pH (e.g., pepsin). Fish pepsins are generally stable at low pH and their optimum activity is in the range of 2.0–4.0 [35]. In this study, during the whole hydrolysis period, the pH and temperature were maintained in the range of 3.3–3.8 and 29–33.9 °C, respectively. Different studies have reported the application of commercial enzymes (e.g., alcalase, flavourzyme, neutrase, papain, and bromelain) to produce protein hydrolysates with a high DH [36–38]. However, Opheim et al. [39] concluded that the addition of papain and bromelain only slightly enhanced DH compared to the hydrolysate produced with only endogenous enzymes (48.6 vs. 48.0%). Also, autolysis is regarded as an economical and simple process to obtain FPH [40].

#### Molecular weight profile.

Electrophoretic analysis was used to visualize formation of lower molecular weight proteins (S1 Fig). The protein pattern of day one (D0) showed mainly fragments with molecular sizes above 50 DA. On the D3, new bands appear in 40 and 30 DA Fig 2B. These bands began to disappear gradually after D5 hydrolysis, except for bands >60 DA. At the end of hydrolysis (D15), the bands >60 DA disappeared while the 40 and 30 DA bands persisted. Several types of proteases (including pepsin, trypsin, chymotrypsin, and collagenase, etc.) are present in fish waste, of which pepsin is the main acid protease in fish viscera with a molecular weight ranging from 27 to 42 DA [35,40]. This may indicate that the persistent bands could be endopeptidases such as pepsin. In addition, bands with molecular sizes below 15 DA predominated during the whole hydrolysis period. The yields of fraction were > 30 DA (13%) and < 30 DA (87%). Potential applications of FPH are wide, covering its use as food ingredients and additives in aquaculture feed, but positive effects depend largely on the molecular weight of peptides [41]. The FPH had a high content in peptides with a molecular weight above 2000 Da (Table 2). Similar values (1–10 DA) was also observed in hydrolysates produced from fish wastes [41]. This molecular weight is advantageous for the discovering of bioactive peptides such as antibacterial, inflammatory, and antioxidant peptides [4,37].

**Table 2:** Proximal composition and molecular weight distribution of hydrolysate from fish waste (dry basis).

	Silage	FPH
**Proximal (g 100g**^**-1**^)		
**Moisture**	80.1 ± 1.61	72.4 ± 0.44
**Protein**	52.8 ± 0.42	61.8 ± 1.48
**Lipids**	11.3 ± 0.75	1.23 ± 0.05
**Ash**	22.2 ± 1.12	17.5 ± 1.27
**NFE (nitrogen free extract)**	13.5 ± 1.64	19.4 ± 1.70
**Molecular weight distribution (Da)**		
**2,120–600**	–	9.5%
**600–255**	–	27%
**255–200**	–	35%
**< 200**	–	29%

### Average yield

Hydrolysis of fish raw material resulted in four fractions after centrifugation: sediment, oil, emulsion, and water-soluble compounds. Fig 1C shows the evolution of fractions distribution during the 5, 10 and 15 days of hydrolysis. The oil content did not change while water-soluble increased and sediment decreased. During hydrolysis, it is generally desired to optimize the hydrolysate yield while minimizing emulsion and sediment fractions [6]. In terms of yield, at the end of the hydrolysis period (D15) the water-soluble fraction (protein hydrolysate) was 67% of the total hydrolysate (dried powder). The hydrolysate yield in D5 was lower than the yield achieved in D10 and D15. The difference in the yield was related to the decrease in sediment content, which is expected, as more proteins in the raw material are cleaved to smaller soluble peptides during hydrolysis and recovered in the soluble fraction, while insoluble materials are recovered in the sediment. This is in accordance with previous reports for other fish hydrolysates [6,42].

### Nutritional composition

The proximate composition of the FPH is shown in Table 2. In terms of moisture content, the FPH contains lower levels than the silage. Ash content, representing the mineral fraction, showed lower content in protein hydrolysate. As expected, the FPH contained a higher protein concentration and lipid low value compared to silage. The protein content is also comparable to other studies, with values which ranged from 60 to 90% depending on which raw material was included for the hydrolysis [6,37,43]. Low lipid content in FPH was expected due to the silage centrifugation to separate insoluble and undigested substances. The low lipid content in protein hydrolysate is generally desirable to reduce lipid oxidation, in fact high-quality hydrolysates are characterized by a high protein content [37], but the content and type of amino acids is usually the primary determinant of nutritional quality of protein. Other authors have also reported comparable results in fish hydrolysates composition [37,43,44].

Table 3 shows free and total amino acids in the FPH. In the present study, we identified seventeen amino acids, encompassing nine essential amino acids (EAA´s; 182 mg g^-1^) and eight non-essential amino acids (NEAA´s; 427 mg g^-1^). At the same time, the free amino acids (FAA´s) content was 8.3% of the total amino acids content. FAA´s contents of 30% were previously reported for protein hydrolysates obtained from waste products of *Gadus morhua* [45]. The FAA`s content depends on the enzymes, the time of hydrolysis, and the DH, as FAA´s is liberated during proteolytic hydrolysis of peptide bonds [6]. It is important to note that taurine was the only amino acid found in a higher concentration in the FAA´s when compared to the total amino acid fraction. It is known that taurine, a free amino acid, is not part of the protein polypeptide chain and hence is found in a greater proportion in the free amino acid fraction [46]. In terms of NEAA´s, FPH contains high amounts of glutamic acid (Glu) and aspartic acid (Asp) 157 and 86 mg g^-1^, respectively. These amino acids contribute to palatability. In fact, *O. maya* diets contain high amounts of these amino acids [19]. Also, Glu plays a critical role in amino acid metabolism because of its role in transamination reactions and is necessary for the synthesis of key molecules, such as glutathione, and Asp is the precursor of methionine (Met), threonine (Thr), isoleucine (Ile), and lysine (Lys) [47].

**Table 3:** Amino acid composition of FPH crab, and squid meal (mg g^-1^ of dry basis) [19]^1^.

	Free	Total	Crab^1^	Squid mantle^1^
**His**	3.9 ± 0.10	12 ± 0.85	22	13
**Arg**	3.5 ± 0.07	49 ± 0.15	65	30
**Thr**	1.1 ± 0.18	24 ± 0.48	53	72
**Val**	1.2 ± 0.02	13 ± 0.21	36	23
**Met**	2.9 ± 0.14	7.0 ± 0.22	28	27
**Lys**	4.4 ± 0.14	45 ± 1.87	80	52
**Ile**	0.9 ± 0.01	11 ± 0.28	39	24
**Leu**	7.4 ± 0.07	14 ± 0.34	71	51
**Phe**	3.0 ± 0.04	80 ± 0.57	36	25
**Asp/Asn**	1.3 ± 0.12	86 ± 1.36	94	63
**Ser**	1.5 ± 0.05	37 ± 0.28	39	28
**Glu/Gln**	4.8 ± 0.78	157 ± 1.68	140	100
**Gly**	2.7 ± 0.07	75 ± 0.74	89	28
**Ala**	3.0 ± 0.31	36 ± 0.35	59	39
**Pro**	0.5 ± 0.08	6.0 ± 1.81	42	16
**Tyr**	1.9 ± 0.26	13 ± 0.35	34	45
**Tau**	7.3 ± 0.49	18 ± 0.64		
**EAAs**	28	182	430	317
**NEAAs**	23	427	497	319

**Fig 3:**
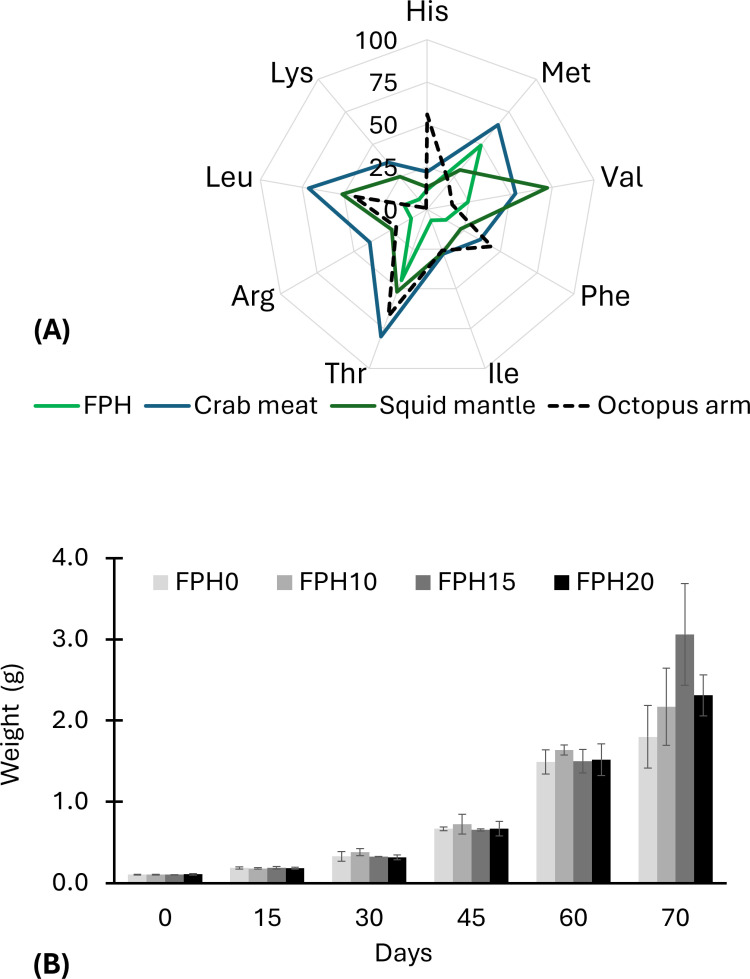
Comparation of essential amino acid (mg g-^1^) of FPH, crab, squid, and octopus arm [19] (A). Growth of *O. maya* juveniles fed FPH diet (B).

Threonine (Thr), histidine (His), leucine (Leu), and Phe (phenylalanine) are EAA´s dominant in the muscle of *O. maya* juveniles [19], therefore play a crucial role for muscle development and these must be obtained through diet to support the high growth rate of cephalopods, see Fig 3. In cephalopod species, Glu, Asp, glycine (Gly), Lys, Leu, and Arg are the six key amino acids from a nutritional viewpoint, promoting growth, regulation of immune systems, and overall health [16,48]. These amino acids represent 70% of the total amino acids in FPH. Furthermore, in octopus it was demonstrated that Phe, Ile, alanine (Ala), Glu, and serine (Ser) are used as metabolic fuel [16,49]. In general, the amino acids are biomolecules that play important roles in cell signaling, nutrient transport, metabolism, and they precursors for synthesis of a wide range of biologically important substances such as phosphatidylserine, sphingomyelin, and cerebrosides, all related to growth and development [50].

The knowledge of FPH composition provided information on nutritional quality and potential nutritive value for *O. maya.* The protein requirements of *O. maya* in captivity, which range between 60–70%, have been effectively met using blue crab and squid meal [19]. Consequently, these ingredients have become crucial in advancing *O. maya* aquaculture. Although FPH is high in protein and low molecular weight peptides (oligopeptides), it contains lower levels of essential amino acids (EAA), such as His, Ile, Phe, Leu, Arg and Thr, compared to crab or squid meal (Fig 3). Therefore, this aspect was considered when designing the experimental diets in this study (crab substitution) and ensuring the essential amino acid requirements for optimal growth of *O. maya*.

### Effects inclusion of protein hydrolysate on octopuses

Results on growth performance and body indices of *O. maya* after *ad libitum* feeding for the experimental diets are shown in Table 4. All diets were well accepted and there was no sign of rejection of the pellets. The octopuses showed clear growth in all groups Fig 3B. However, the highest final weight (p < 0.05) was detected in FPH15 fed octopuses; indeed, weight was 29-fold of the initial body weight. By the end of the experiment, significant differences in survival rate were observed among the diets (Table 4). It is noteworthy that a higher survival rate was found in the treatment FPH15 (90%), whereas the octopuses fed as FPH0 showed the lower survival rate (72%). Indeed, a 10% decline in survival was observed in octopuses fed FPH15 diet at 60 days. These results are in line with our previous study in octopus, in which survival was related to silage-based diets (unpublished data). Weight gain and SGR showed significant differences among experimental diets (p < 0.05). Octopuses fed with FPH15 had the highest WG and SGR compared to other treatments (Table 4). However, octopuses fed with diets containing the highest level of FPH (20%) had the lowest values of WG. These results are in accordance with previous studies in *Dicentrarchus labrax* [51], *L. vannamei* [11], and *Rhamdia quelen* [52], where the moderate inclusion of FPH had positive effects on nutritional indices and immune parameters leading to better gut health and survival rate. In *O. maya*, several studies have shown that the formulated diets using crustaceans and squid meals are still better compared to alternative protein sources [19–23]. Aguila et al. [21] found lower growth rate (SGR, 0.86% day^-1^) in *O. maya* fed the diet with soluble fish protein concentrate (CPSP: 0, 5, 10, 15, and 20%) against a crab diet (SGR, 3.71% day^-1^). Also, Martínez et al. [22] reported marginal (0.36% day^-1^) and negative (−0.73% day^-1^) SGR values in octopuses fed a silage-based diet.

**Table 4:** Biological parameters in *O. maya* fed formulated diets with protein hydrolysate from silage fish waste.

Items	FPH0	FPH10	FPH15	FPH20	*p-value*
**IW (g)**	0.103 ± 0.004	0.104 ± 0.003	0.103 ± 0.001	0.109 ± 0.009	0.387
**FW (g)**	1.80 ± 0.358	2.17 ± 0.475	3.06 ± 0.780	2.31 ± 0.253	0.001
**WG (g)**	1.70 ± 0.690	2.06 ± 0.470	2.95 ± 0.800	2.20 ± 0.380	0.001
**SGR (% day**^**-1**^)	3.99 ± 0.571	4.32 ± 0.290	4.76 ± 0.350	4.32 ± 0.130	0.003
**SR (%)**	72.2 ± 9.6	83 ± 17	90 ± 9.0	84 ± 1.37	0.343
**Somatic indices**					
**HSI (%)**	5.13 ± 0.139	5.43 ± 0.083	5.82 ± 0.679	5.50 ± 0.648	0.149

Initial weight (IW), Final weight (FW), Weight gain (WG), Survival rate (SR), and Hepatosomatic index (HIS).

Generally, protein hydrolysates are composed of small fragments of peptides, typically containing 2–20 amino acids [37]. They have quicker absorption and digestion rates than intact proteins [41]; as a result, can be rapidly absorbed by intestinal cells and enter the body more rapidly than larger nutrients, potentially generating signals that stimulate appetite. Indeed, certain free amino acids (such as Leu, Glu, Lys, and Tau) are known to have chemo-attractive properties for olfaction and/or ingestion in carnivorous species [52]. Le Bihan et al. [13], used marine by-product silage as a diet enrichment for feeding juvenile cuttlefish, the results show that peptide-enriched diets can improve growth and feed conversion ratio compared to non-enriched diets. They also point out that the fish silage contains mainly small peptides (< 6.5 DA), which can possess bioactive properties. Bioactive peptides are amino acid chains derived from proteins that, in addition to providing nutritional benefits, perform specific biological functions in the body. These molecules are characterized by having a molecular weight within the range of 0.2 to 2.0 DA [4,37]. In the present study, higher growth performance obtained with FPH inclusion may potentially be attributed to bioactive components in the hydrolysate such as small peptides (< 2.12 DA, Table 2); these nutrients could play a crucial role in enhancing diet taste and palatability and consequently its consumption. However, the relationship between small peptides and physiological benefits remains unclear.

The effects of diets on digestive enzymes activity are shown in Table 5. The study of digestive enzymes contributes to a better understanding of the digestion and assimilation of formulated feeds [53]. No significant differences in acid enzymes activities were observed among octopuses fed all experimental diets. These results agree with those previously reported for *O. maya* [19,21–23,54]. Protein hydrolysates have been studied in several aquatic animals, particular in fishes [4,10–12,55]; consistently, the results suggest that dietary inclusion of protein hydrolysates can stimulate protease activity and increase digestibility of protein. Also, they suggest that FPH contains peptides with the ability to stimulate production of insulin-like growth factors which may enhance fish growth. The positive effects recorded seem to depend on the rate of inclusion, usually an appropriate level [4,41]. In the current study, acid enzymatic activity showed an increase trend from lowest to highest inclusion levels, meanwhile, the alkaline enzymatic activity decreased. Similar findings were reported by Aguila et al. [21] who reported that general proteases and trypsin activity in the diets of wild octopuses (*O. maya*) fed with 15% CPSP were significantly higher, and lower values in octopuses fed 20% CPSP. The decrease in alkaline activity can be explained by the fact that the proteins in the FPH are already partially or fully digested. In this sense, the low molecular weight peptides in FPH may have reached the intestine faster, with a consequent decrease in secretion of alkaline protease. Research conducted by Nikoo et al. [55] showed that trypsin and pepsin activity in *Sparidentex hasta* and *Acanthopagrus arabicus* juveniles fed with FPH diets were lower than the control treatment, which may be due to the lack of metabolic energy expenditure to secret digestive enzymes by fish due to the high digestibility and absorption of di-, tri- and oligopeptides in FPH.

**Table 5:** Enzymatic activity and glycogen in *O. maya* fed formulated diets with protein hydrolysate. Mean values ± SD. (n = 9).

Items	FPH0	FPH10	FPH15	FPH20	*p-value*
**Acid (U mg protein**^**-1**^)	28382 ± 3180	27826 ± 4229	29310 ± 3110	30389 ± 4107	0.312
**Alkaline (U mg protein**^**-1**^)	5277 ± 145	5637 ± 492	4750 ± 230	3550 ± 455	0.001
**Glycogen digestive gland (mg g**^**-1**^)	3.84 ± 0.670	3.36 ± 0.776	3.16 ± 0.365	3.13 ± 0.169	0.226
**Glycogen muscle (mg g**^**-1**^)	0.330 ± 0.032	0.572 ± 0.094	0.535 ± 0.010	0.570 ± 0.07	0.004

On the other hand, higher HSI values were observed in octopuses fed with FPH15 compared to FPH0. In general, HSI is used as indicators of physiological condition and nutritional status [19]. A higher HSI could indicate that octopuses have a better capacity to digest and store nutritional reserves such as glycogen in the digestive gland. However, the glycogen content (3.84 mg g^-1^) was high (p < 0.05) in the digestive gland of octopuses fed FPH0 compared to the other diets (average, 3.22 mg g^-1^). Glycogen in arm muscle (average, 0.55 mg g^-1^) was high (p < 0.05) in octopuses fed diets with FPH (Table 5). Rosas et al. [20] reported that muscle glycogen concentration was affected by the type of diet, with high values in animals fed clam (> 2 mg g^-1^) compared with the fresh crab (< 1.5 mg g^-1^). In general, glycogen content obtained here agrees with those previously reported for wild and farmed octopuses [19,21,22,54]. Research indicates that *O. maya* juveniles with higher SGR have higher glycogen content in the arm muscle [19,22]. In *Paroctopus digueti*, the increasing glycogen content in the arm muscle reflects better use of the prey captured and therefore better body condition [56]. Arg is among the most metabolically active amino acids in cephalopods [50] and is highly used with source of glycogen production. In this sense, the Arg content could affect glycogen levels in octopus tissues fed protein hydrolysate diets, in fact the Arg content in FPH is lower compared to crab and squid meal Fig 2.

Finally, this study focused on the use of FPH as a component in diets for juvenile octopus, since feed costs in *O. maya* aquaculture can represent a significant portion of the costs of production ≈50% (personal communication); the average price of fresh crab is ≈3 USD/kg respectively and may vary depending on factors such as regional availability. In this context, the use of FPH-based foods can improve the viability of *O. maya* aquaculture. The use of *H. plumierii* by-products to produce FPH is a practice that promotes a circular economy by closing the material cycle. The creation of small hydrolysis units for FPH production in coastal communities could generate economic benefits for small-scale fishers and processors, reduce pressure on blue crab, and decrease octopus feed costs.

## Conclusion

This study demonstrates the nutritional potential of a fish by-product hydrolysate with a high protein content (61.8%) but a low concentration of essential amino acids (182 mg g⁻¹). Molecular weight distribution profiling of FPH demonstrated that 85% of the protein content comprised a peptide mixture, with molecular weights below 2.12 DA. Our study demonstrated that replacing up to 15% of crab meals in the diet of *O. maya* with FPH, does not compromise growth performance and survival. Hence, incorporating FPH into diets formulation may offer unique advantages in overall physiological well-being for *O. maya*; this is promising for the future of octopus farming. Although the current findings are promising, it is necessary to conduct studies aimed at determining the most appropriate level of inclusion of FPH in feeds and analysis of nutritional profiles in octopuses. Finally, the use of FPH provides a good option for increasing circularity and sustainable waste management solutions for the fishery industry.

## Supporting information

S1 Fig Image is an uncropped gel image corresponding to protein pattern Fig 2B. All lanes are marked in accordance with labelling in the manuscript and any unused lanes are marked with an X. Lane MW: protein marker; DA: kilodalton; lane 0, 3, 5, 8, 10, and 15 days of hydrolysis. (TIF)
