# Supplementary figures and images for: Protein hydrolysates from fish wastes: nutritional characteristics and its inclusion in diets for Octopus maya
Source: PLoS One. 2025 Apr 18;20(4):e0321572. doi: 10.1371/journal.pone.0321572 (PMC12007706; doi:10.1371/journal.pone.0321572)

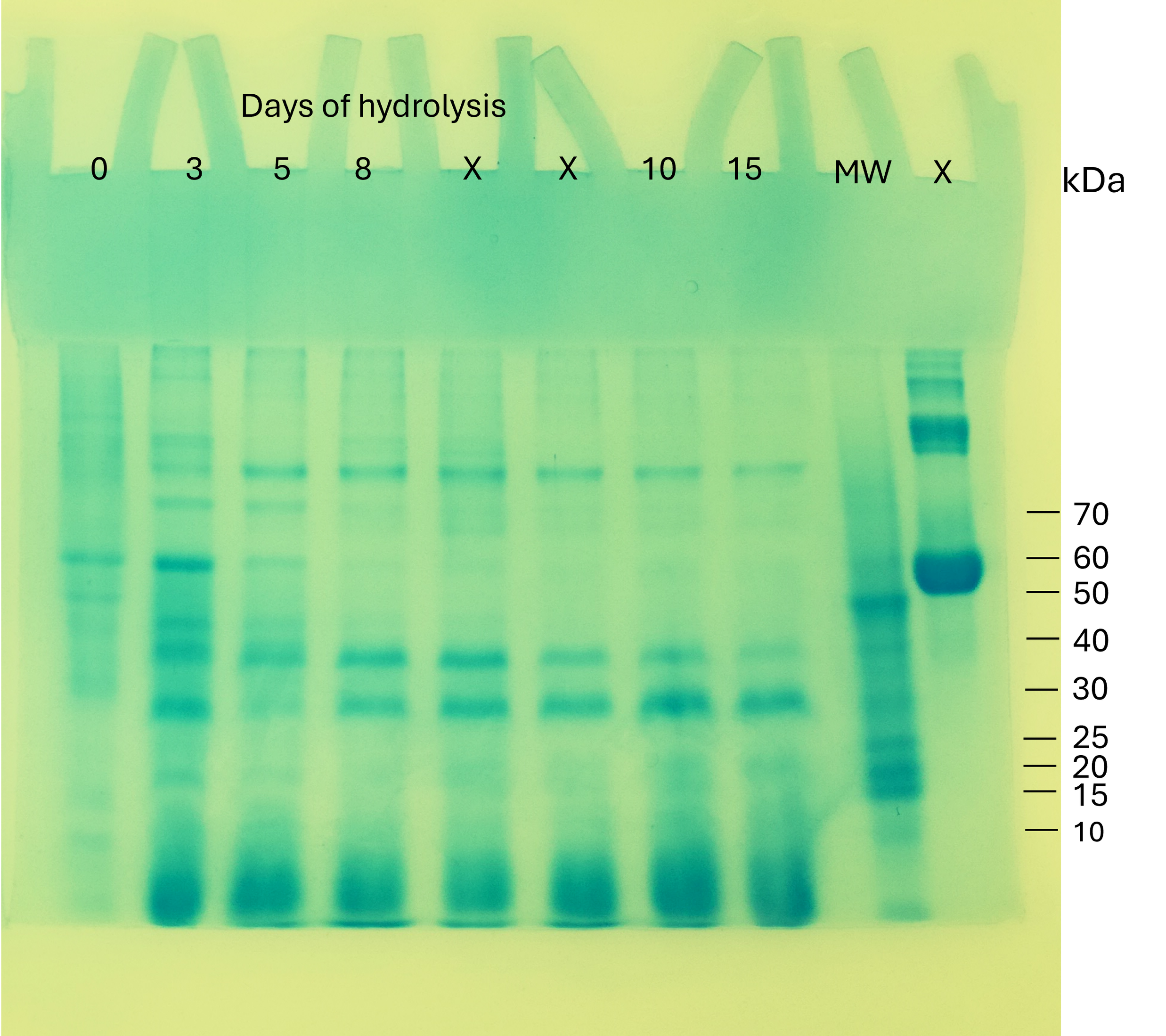

Supplement: S1 Fig — (TIF) [file pone.0321572.s001.tif]
